# Measuring changes in human tumour vasculature in response to therapy using functional imaging techniques

**DOI:** 10.1054/bjoc.2001.2077

**Published:** 2001-10

**Authors:** H Anderson, P Price, M Blomley, M O Leach, P Workman

**Affiliations:** CRC PET Oncology Group, MRC Cyclotron Unit,Department of Imaging, Faculty of Medicine, Imperial College School of Medicine, Hammersmith Hospital, Du Cane Rd, London, W12 0NN; MRI Unit, Royal Marsden Hospital, Sutton, Surrey; CRC Centre for Cancer Therapeutics, The Institute of Cancer Research, Block E, 15 Cotswold Road, Belmont, Sutton, Surrey, SM2 5NG, UK

## Abstract

Antiangiogenic and antivascular agents provide new approaches to treating tumours. These may avoid many of the problems experienced with current approaches such as inherent and acquired resistance to treatment. Tumours do not grow beyond 1–2 mm^3^in size without the development of new vessels (Folkman, 1971). Such neo-vascularization (angiogenesis) allows tumour cells to increase their nutrient supply, survive and proliferate despite the new vessels often having structural and functional differences compared to normal tissue vasculature. Treatments targeted at tumour vasculature have produced impressive results in animal models (Lindsay et al, 1996; Watson et al, 1996; O’Reilly, 1997; Horsman et al, 1998). These therapies are now entering clinical trials. However, the successful introduction of these therapies into clinical practice will require the development of reliable ways to assess angiogenesis and its modification or inhibition in vivo. Here we discuss some of the emerging imaging techniques that may be useful. © 2001 Cancer Research Campaign  http://www.bjcancer.com


*British Journal of Cancer* (2001) **85**(8), 1085–1093
© 2001 Cancer Research Campaign
doi: 10.1054/ bjoc.2001.2077, available online at http://www.idealibrary.com on IDEAL

Editorial

### Measuring changes in human tumour vasculature in response to therapy using functional imaging techniques

**H Anderson[1], P Price[1], M Blomley[2], MO Leach[3,4] and P Workman[4] for the Cancer Research Campaign PK/PD Technologies Advisory Committee**

[1]CRC PET Oncology Group, MRC Cyclotron Unit & [2]Department of Imaging, Faculty of Medicine, Imperial College School of Medicine, Hammersmith Hospital, Du Cane Rd, London W12 0NN; [3]MRI Unit, Royal Marsden Hospital, Sutton, Surrey; [4]CRC Centre for Cancer Therapeutics, The Institute of Cancer Research, Block E, 15 Cotswold Road, Belmont, Sutton, Surrey SM2 5NG, UK

**Summary** Antiangiogenic and antivascular agents provide new approaches to treating tumours. These may avoid many of the problems experienced with current approaches such as inherent and acquired resistance to treatment. Tumours do not grow beyond 1–2 $mm^3$ in size without the development of new vessels (Folkman, 1971). Such neo-vascularization (angiogenesis) allows tumour cells to increase their nutrient supply, survive and proliferate despite the new vessels often having structural and functional differences compared to normal tissue vasculature. Treatments targeted at tumour vasculature have produced impressive results in animal models (Lindsay et al, 1996; Watson et al, 1996; O'Reilly, 1997; Horsman et al, 1998). These therapies are now entering clinical trials. However, the successful introduction of these therapies into clinical practice will require the development of reliable ways to assess angiogenesis and its modification or inhibition in vivo. Here we discuss some of the emerging imaging techniques that may be useful. © 2001 Cancer Research Campaign http://www.bjcancer.com

Tumour blood supply is complex. The tumour phenotype does not have the normal arteriole/capillary/venule complex. Significant shunting via anastomotic vessels and deranged vasculature are common features. The capillary bed responsible for nutrient supply in normal tissue comprises only about 5% of the total tissue vasculature (Jain, 1988). In tumours this can be less and in addition, not all the capillary bed functions efficiently. Thus, in tumours despite apparently good blood supply, the all-important capillary bed is often deficient, leading to tumour hypoxia and necrosis.

Antiangiogenesis therapy is aimed at inhibiting new endothelial cell development to prevent tumour growth and metastasis. Antivascular therapy directly targets existing endothelial cells leading to destruction of the tumour vasculature, which will then result in tumour cell death.

The effects of antivascular agents may be the easier to define. The direct consequence of destroying a tumours vascular supply would be a reduction in the blood flow. Combretastatin A4 Phosphate an antivascular agent in animal models has been shown to acutely reduce the blood flow to tumours in vivo (Anderson et al, 2000). The accurate and reproducible measurement of blood flow seems a reasonable measure for this type of agent. Indirect measures of the effects of vascular shutdown may also be useful in assessing the more chronic effects of these agents, for example, changes in extent or degree of hypoxia.

The effects of antiangiogenic agents may be more difficult to determine. Most antiangiogenic agents have an inhibitory effect i.e. they work by preventing the formation of new blood vessels. They do this by a wide range of mechanisms that may produce different effects in the tumour at different time points. For example, an anti-VEGF agent may produce early changes in vascular permeability, followed by changes in the interstitial pressure of a tumour with later reduction in the number of new blood vessels formed. The exact pathophysiological endpoints of the various antiangiogenic approaches and how they are best assessed needs to be identified. In vivo animal data prior to clinical assessment may provide a guide to the anticipated physiological changes.

*Received 6 November 2000*
*Accepted 15 August 2001*

*Correspondence to:* P Price,Academic Department of Radiation Oncology, Christie Hospital, Manchester M20 4BX;
E-mail: anne.mason@christie-tr.nwest.nhs.uk

### CURRENT ASSESSMENT METHODS

At present the most widely used static, invasive method to assess human solid tumour angiogenesis is quantification of intratumoral microvessel density (IMD) in biopsy material. Antibodies binding to endothelial cell markers are quantitated by immunohistochemical techniques. The degree of IMD is thought to reflect the intensity of tumour angiogenesis. A correlation between IMD and angiogenic factor expression, tumour growth, the occurrence of distant metastasis and prognosis have been reported (Gasparini, 1996). IMD is currently considered the gold standard for histological assessment of the degree of angiogenesis within a tumour. Measuring change in IMD in response to therapy requires repeat biopsy. Other surrogate markers for changes in angiogenesis have been suggested, such as changes in plasma/urinary levels of VEGF or changes in endothelial proliferation markers such as CD105 (endoglin), a receptor for transforming growth factor (TGF) in vascular endothelial cells which is highly up-regulated in blood vessels of tissues where neovascularisation occurs (Crew et al, 1999; Davies et al, 2000).

Current clinical trials of the new antiangiogenesis therapies have incorporated a number of endpoints in an attempt to look for a correlation of various measures of endothelial cell damage/turnover with a suggestion of activity. For example, Wamil et al (1997) used soluble E selectin levels to measure the effects of CM101, an antineovascular agent as proof of endothelial engagement in an inflammatory process.

However, there is as yet no one definitive measure of the effect of antiangiogenic or antivascular agents in man.

One emerging approach to measure therapy-induced changes is to use repeatable, non-invasive methods of assessment. Magnetic resonance imaging (MRI), Doppler ultrasound (U/S), computed tomography (CT), positron emission tomography (PET), angiography and spectroscopy are being explored to measure a variety of relevant parameters: vascular density, blood flow, blood volume, permeability and combinations of these parameters. This paper will discuss the relative merits of each and future direction of imaging angiogenesis and vascular parameters.

### MRI

A number of MRI techniques are being developed that could be used to measure tumour vascular changes.

### *Dynamic contrast-enhanced MRI*

Here, i.v. injected contrast is used to image capillary function (see Figure 1). MR contrast agents principally use gadolinium chelates, such as DTPA, which provide good contrast between malignant tumours and surrounding tissues. The strongly paramagnetic gadolinium is not observed directly, but it has the property of relaxing or restoring the intrinsic MR signal of the water molecules that continually approach the gadolinium. This provides a very large amplification effect, as one molecule can affect very many water molecules. The higher uptake of DTPA in tumours compared with normal tissues and many benign lesions results from vascular factors (increased perfusion, permeability) and frequently a higher extracellular volume.

During the last 7 years there has been considerable interest in dynamic imaging techniques, using examination of contrast uptake and washout parameters. The method has high sensitivity and combining patterns and the shape of the uptake curve increases the diagnostic specificity (Heywang et al, 1989; Flickinger et al, 1993; Kaiser, 1993, 1994; Boetes et al, 1994; Orel et al, 1994; Fobben et al, 1995; Kerslake et al, 1995; Perman et al, 1996). Typical measurements include the maximum signal increase, rate of signal increase and time to maximum signal (Konig et al, 1990; Verstraete et al, 1994; Hawighorst et al, 1998).

Quantitative imaging sequences and modelling approaches have been developed to calculate uptake parameters, transfer rates, and extracellular volume (Tofts and Kermode, 1991; Su et al, 1994; Hoffmann et al, 1995; Parker et al, 1997; Hawighorst et al, 1998; Weissleder et al, 1998) The MR community is currently working to standardise such parameters (Tofts et al, 1999). The interpretation of these values requires validation. We need to know if these surrogate measures using gadolinium uptake into tissue directly relate to physiological processes. Preliminary preclinical and clinical work suggests that they do. Correlative studies have compared these parameters with histopathological findings (Hulka et al, 1997; Hawighorst et al, 1998; Postema et al, 1998) and with clinical outcomes such as response to radiotherapy (Mayr et al, 1996) or prognosis (Hawighorst et al, 1998; Weissleder et al, 1998). From these studies it is becoming clear that the enhancement parameters measured are composite and represent a combination of (1) vessel density, (2) permeability, (3) character of the extravascular space and (4) perfusion. Correlation with specific physiological factors is now required.

The next step needed is assessing how changes in these parameters reflect changes in vascular physiology in response to therapy. There are 2 major methodological problems. First, the entry of gadolinium into tissue is not linearly related to blood flow and perfusion. Secondly, as yet, no methodology exists to measure the input function (the measure of gadolinium delivery to the region of interest). A ‘standard’ input function is often included for modelling purposes, but for repeat measurements this may be totally inadequate, particularly if the antivascular therapy itself changes the input function, e.g. by changing the cardiac output. Until methodology can measure this input function the technique can only be semiquantitative and changes in measurements in response to therapy must be interpreted with caution.

### *Macromolecular contrast media*

The rapid diffusion of low molecular weight contrast agents such as gadolinium DTPA complicates interpretation of their uptake kinetics. One attempt to simplify the analysis has been the development of macromolecular MR contrast media (MMCM). Agents such as polylysine-Gd-DTPA and albumin-gadolinium DTPA have been used. These agents diffuse very slowly, if at all, through normal endothelial barriers and are well suited to define the hypervascularity and hyperpermeability inherent in tumour microvasculature (Brasch et al, 1997). MMCM can provide a quantitative measurement of blood volume and permeability, expressed as the permeability surface area product (PS) (Kuwatsuru et al, 1993; Shames et al, 1993; Brasch et al, 1997). Brasch et al (1997) have used this technique to monitor the effects of antiVEGF antibody on athymic rats grafted with a human breast carcinoma. These agents are not yet approved for clinical use due to incomplete elimination and potential immunogenicity (Roberts, 1997), limiting this promising technique to animal studies.

### *Perfusion and diffusion imaging*

When a bolus of paramagnetic contrast (such as Gd-DTPA) passes through the capillary network, there is a marked magnetic susceptibility mismatch between the blood and the surrounding extracellular space, resulting in a pronounced drop in signal seen on gradient echo images. The signal drop depends on the density of small capillaries and the concentration of contrast in the capillary bed, providing the basis for measuring regional blood volume (rBV) (Villringer et al, 1988; Rosen et al, 1990; Aronen et al, 1994; Boxerman et al, 1995). In the brain, echo planar imaging techniques are commonly employed, with sensitivity to capillary dimensions above 3 μm radius, and thus include contributions from major vessels. Spin echo sequences are only sensitive to small capillaries, with response peaking between 3 and 7 μm (Fisel et al, 1991; Boxerman et al, 1995), providing a better assessment of microvascular volume and nutritive perfusion. These approaches have largely been confined to cerebral measurements and in the absence of measured input functions provide relative measures rather than absolute perfusion.

### *Oxygen-dependent MR imaging*

Blood-oxygenation-level-dependent (BOLD) MRI was originally developed for use in brain activation studies (Ogawa et al, 1990;

 *© 2001 Cancer Research Campaign*

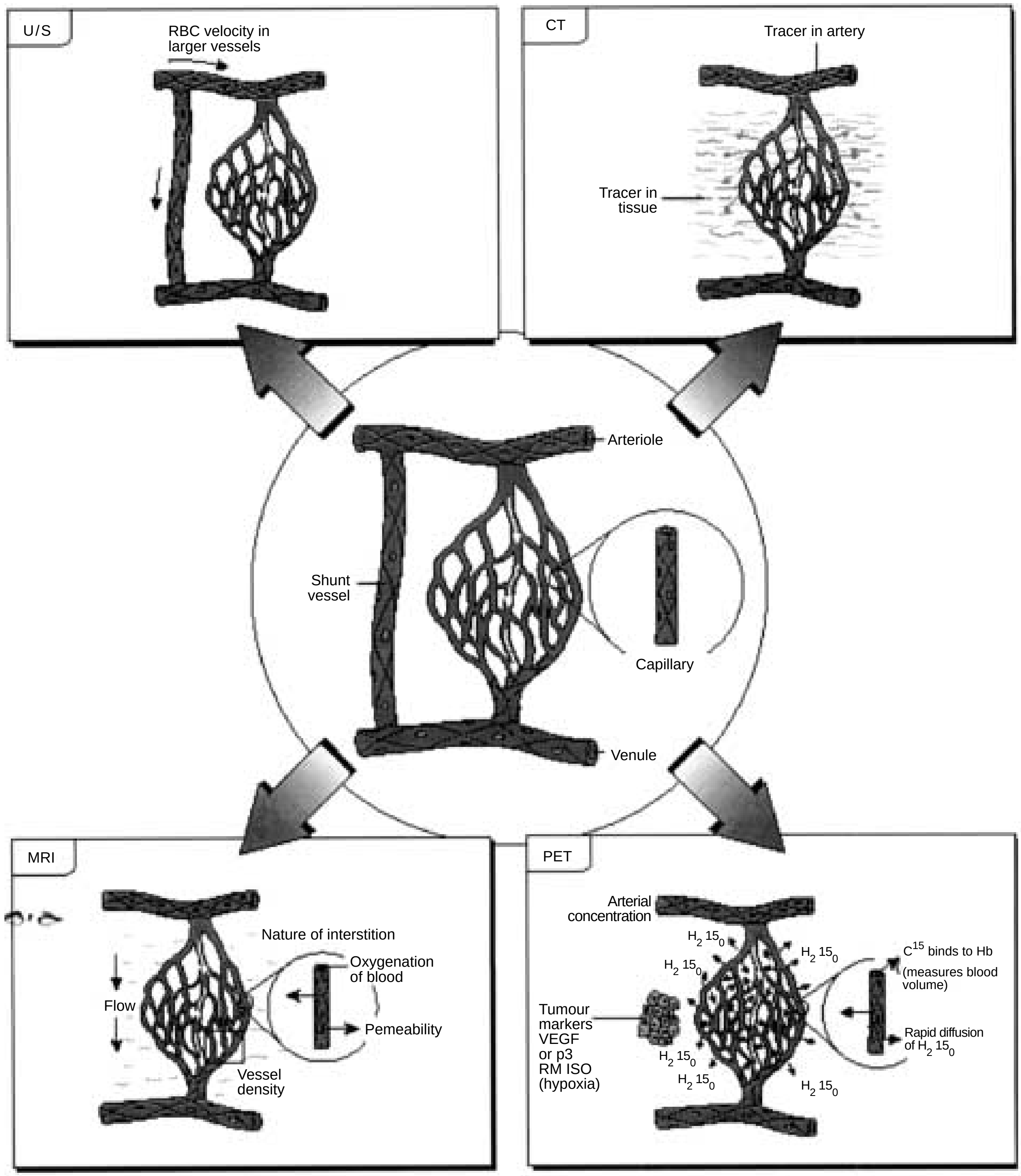

**Figure 1** Illustration of a segment of tumour vasculature showing how various imaging techniques, Doppler ultrasound (U/S), computerised tomography (CT), magnetic resonance imaging (MRI) and positron emission tomography (PET) use aspects of tracer and physiological behaviour to derive their measured parameters van Zijl et al, 1998). Changes in the concentration of deoxyhaemoglobin change the $T_2$-weighted signal, acting as an intravascular contrast agent for MRI. This is the basis of functional MRI (fMRI) for use in brain activation studies. Physiological change such as blood flow modification, or other mechanisms affecting the degree of desaturation of oxygen in the blood within the tissue can thus be

*© 2001 Cancer Research Campaign* 

imaged. BOLD-type MRI changes have now been reported following changes in blood flow and oxygenation in the heart (Reeder et al, 1996) and in tumours (Karczmar et al, 1994; Robinson et al, 1995). The signal will depend on a combination of vessel density, blood oxygenation, blood volume and flow, but changes have not been directly correlated with basic physiological parameters (van Zijl et al, 1998).

### MR angiography

Techniques used in MR angiography are based on phase contrast methods (Wedeen et al, 1985; Dumoulin and Hart, 1986), time of flight (1994; Axel, 1986; Atkinson et al) or contrast enhanced methods (Kouwenhoven, 1997). The first 2 techniques image larger vessels, but the latter technique is also able to demonstrate general contrast changes arising from uptake in the capillary bed. They are associated with the use of subtraction techniques (for contrast or spin-labelled studies), 3D-imaging techniques and 3D display using maximum intensity projections. Their main role would be in evaluating the tumour's supporting vasculature, but contrast-enhanced measurements may also provide qualitative information on change in the capillary bed. There is no work yet published on this technique being used to assess response to antiangiogenic or antivascular therapy.

### Magnetic resonance spectroscopy (MRS)

MRS is able to assess tumour vasculature indirectly by evaluating the molecular composition of tissue and changes therein. The various techniques of $^{1}H$, $^{13}C$ and $^{31}P$ NMR measure the distribution and proportion of molecules within the tissue reflecting such factors as tissue bioenergetics, pH, phospholipid membrane turnover, oxygenation and lactate levels within the tumour (Stubbs, 1999). These factors can be semi-quantitated and followed serially allowing the effects of therapeutic manipulations to be monitored. The technique has been used extensively in animal models looking at the effects of blood flow modifiers such as nicotinamide and carbogen (Robinson et al, 2000), hydralazine (Nielsen et al, 1999) and vascular occlusion (McCoy et al, 1995). One of the major problems of MRS is lack of sensitivity especially in deep-seated human tumours. Despite the promising results in animal models this technique has not yet been developed to assess vascular therapy in man.

### MR methodology developments required

For MRI techniques to be able to measure physiological changes in response to antiangiogenic/antivascular effects several developments are needed.

1. Correlation of measurements with underlying pathophysiology is required. More work is needed to validate the biological interpretation of many of the parameters being measured.
2. Standardised evaluation and modelling techniques are required. Approaches used to evaluate permeability and blood flow vary widely, using different measurement methods and analysis techniques. Generally, input functions are not employed, and bias introduced by departure from the model's assumptions are not explored or corrected for. There is a need to establish a consensus as to the appropriate methodology, with the necessary quality assurance and validation procedures. The importance of methodology for measuring the arterial input function needs to be established.
3. Reproducibility studies are required. Studies assessing the reproducibility of the measurements, serial monitoring of changes and correlating change in MR phenomena with change in pathophysiology are needed. Without test/retest data, interpretation is unreliable.
4. Co-registration. Co-registration of images for comparison of signal before and after therapy is difficult. Current techniques can only provide high-quality dynamic information from a small number of sections e.g. 3 per tumour. These sections have to be identically placed on repeat studies in order for the qualitative comparison to be enacted on a pixel by pixel basis. Increasing speed of instrumentation is leading to the ability to perform full volume 3D studies with acceptable temporal resolution.

### Summary

The potential strength of dynamic contrast-enhanced MRI may lie in its ability to provide a composite high-resolution snapshot of the microvasculature. Tumour vasculature is characterised by a dynamic and heterogeneous pathophysiology with changes in vessel density, permeability and interstitial pressures. Anti-angiogenic and antivascular therapies are likely to have effects on several aspects of the vasculature. MRI is available in many centres. However, validation of the physiological correlates and methodology developments is still required. It is likely that MR techniques will be used in the future as a general broad measure of the changes in vasculature.

### Doppler ultrasound

Doppler U/S techniques can determine the presence of blood flow, its velocity and in certain circumstances estimate intratumoral flow resistance (Delorme and Knopp, 1998) (see Figure 1). Colour Doppler has overtaken earlier Spectral Doppler scanning in tumour imaging. The frequency shift is proportional to the velocity of the red blood cells within the measured vessels after allowing for differences in the angle of the ultrasound and vessel. The velocity of the red blood cells within the measured vessels determines the frequency. Power Doppler is a form of colour Doppler that encodes the power in the Doppler signal in colour. The power it detects depends on the amount of blood present (Martinoli et al, 1998). Power Doppler has advantages over colour Doppler, including increased sensitivity to flow detection and improved detail. A number of different Doppler parameters can be measured by the various Doppler methods. These include pulsatile index, resistive index (RI), acceleration index (AI) and peak systolic flow velocity ($V_{max}$). As well as differentiating different patterns of flow e.g. pulsatile flow, constant flow, and triphasic flow. Recently, complex computer-assisted image analysis methods are attempting to quantify information from colour Doppler studies (Delorme and Knopp, 1998).

The patterns of Doppler signal have been correlated with a diagnosis of malignancy in some tumours (Wells et al, 1977; White and Cledgett, 1978; Mountford and Atkinson, 1979; Burns et al, 1982).

Validation studies to correlate U/S parameters with physiology have been undertaken. U/S parameters are higher in some tumours

 *© 2001 Cancer Research Campaign*

and correlated with histological assessment of vascularity as measured by IMD but this was not found in all studies (Sterns et al, 1996; Emoto et al, 1997; Peters-Engl et al, 1998).

Development of microbubble contrast agents which can be given intravascularly to enhance signals from vessels and may also have tissue specific properties. Microbubbles increase both grey-scale and Doppler signals from vessels both by increasing backscatter but at higher acoustic powers by resonating and producing harmonic microbubble-specific signals (Harvey et al, 2000). At even higher power bubble disruption and destruction occurs. Small studies suggest that the use of such microbubbles can increase the sensitivity of detection of liver metastases (Harvey et al, 2000). In addition, the latter property of the bubbles i.e. their destruction is being manipulated in attempts to measure tissue blood flow: their subsequent reappearance and concentration in tissue allows the calculation of blood flow and potentially could be used as a measure of tumour blood flow (Aronson et al, 1993; Wei et al, 1998).

### *Limitations*

1. Doppler methods are not able to assess the microvasculature. Macrovasculature may correlate with microvasculature in some instances; tumours well endowed with larger vessels have corresponding rich small vessel supply, but U/S does not as yet provide an accurate measure of the microvasculature. New developments with microbubble techniques have the ability to increase the sensitivity to capillary blood flow but are in the early stages of development.
2. Flow velocities under 1 cm $s^{-1}$ are difficult to detect due to artefacts caused by tissue movement (Delorme and Knopp, 1998).
3. U/S results depend on the exact positioning of the probe. Reproducible measurements of the same slice of tissue are difficult.
4. Attenuation is exponential while ultrasound displays are inherently nonlinear.

These limitations mean that although simple and convenient, it may not be the best technique to assess changes after anti-angiogenic/antivascular therapies.

### Positron emission tomography (PET)

### *Blood flow and volume techniques*

Positron emission tomography is a powerful tool in the area of vascular research. In the 1970s and 1980s techniques were developed utilizing $^{15}$O-labelled $H_2{}^{15}O$ and $C^{15}O$ to quantitate regional blood flow and volume, and also oxygen utilization in the brain and heart (Lammertsma et al, 1990; Araujo et al, 1991). It has subsequently been modified and used to measure blood flow and exchanging water space in breast tumours (Wilson et al, 1992). In a perfusion scan, $H_2{}^{15}O$ is administered by an i.v. infusion to a patient centred in the PET scanner. To calculate flow, 2 measurements need to be made: 1. tissue time concentrations which can be determined quantitatively from the PET signal and 2. arterial concentration which is measured by arterial sampling using a cross calibrated well counter. Perfusion in ml $min^{-1}$ $ml^{-1}$ of tissue and fractional volume of distribution (VD) are calculated using the PET operational equation for perfusion.

The main advantage of this technique is that $^{15}$O has several fundamentally useful characteristics as a flow and volume tracer: it is biologically inert, chemically stable with no physiological effects and its short half-life allows rapid and repeat studies to be performed. Regional tumour blood volume (BV) can also be measured (Lammertsma et al, 1983; Leenders, 1994). $C^{15}O$ binds to haemoglobin to form deoxyhaemoglobin, which remains within the vascular space and therefore provides a measure of intravascular volume. The collection of data with an input function allows a quantitative, non-invasive, repeatable measure of tumour perfusion with this technique. Wilson et al (1992) refined the technique to measure perfusion and volume of distribution in breast tumours. However, despite being regarded by many as the gold standard for non-invasive measurement of perfusion very little work has been published investigating perfusion in tumours outside the CNS. One study that has been performed already mentioned using PET $H_2{}^{15}O$ and $C^{15}O$ techniques to assess the effects of Combretastatin A4 Phosphate and investigating its mechanism of action (Anderson et al, 2000).

### *Other tracers of use in PET*

PET has the major strength within the imaging modalities of being able to measure metabolic and biochemical processes in vivo. It uses radionuclides of commonly occurring elements such as oxygen, carbon and nitrogen, which can be incorporated into a wide range of compounds. In addition, it can measure down to picomolar concentrations in a dynamic and repeatable way.

Labelling of specific markers of tumour vasculature and angiogenesis for analysis by PET has considerable potential. Radiolabelled antibodies to VEGF are in the process of being developed. VEGF mRNA is up-regulated in virtually all human tumours so far examined (Ferrara, 1996). Correlations have been observed between VEGF protein expression and microvessel density (Toi et al, 1994). There are numerous other molecules involved in the angiogenesis process that could potentially be targets for PET labelling e.g. bFGF, $\alpha_v\beta_3$ integrin (Haubner et al, 2001), E-selectin, angiostatin and matrix metalloproteases.

Indirect assessment of tumour vasculature can also be performed using markers of tissue hypoxia. Nitroimidazoles for example, are a class of compounds that undergo intracellular metabolism depending on the availability of oxygen in the tissue. At biologically significant levels of hypoxia nitroimidazoles are trapped in cells. $^{18}$F-labelled fluoromisonidazole has been used to image hypoxia in human tumours in vivo (Koh et al, 1992; Rasey et al, 1996) and has been used to monitor the effects of conventional therapy (Koh et al, 1995).

### *Limitations*

1. PET availability is currently limited.
2. Resolution is limited and definition of regions of interest can be difficult without co-registration with CT or MRI.
3. Scans require arterial cannulation to obtain an accurate input function.

In summary, PET is currently the gold standard in quantitating tumour perfusion, fVD and blood volume. Its absolute measurement of separate measures lends itself to not just being able to measure P/D endpoints but also investigating mechanisms of action of antiangiogenic and antivascular agents.

### CT scanning

As with MRI the development of powerful machines capable of rapid dynamic analysis has led to the introduction of a CT

*© 2001 Cancer Research Campaign*

**Table 1** Summary of the various imaging techniques available to assess aspects of tumour vasculature

| Technique | Measured parameters | Pathophysiological correlates | Advantages | Disadvantages |
|---|---|---|---|---|
| Contrast-enhanced MRI | Maximum uptake<br>Rate of uptake<br>Transfer rates<br>Extra-cellular volume<br>Relative blood volume<br>Relative perfusion | Vessel density<br>Permeability<br>Perfusion<br>Extravascular space | Availability<br>High spatial resolution<br>Registered anatomy | Image quality can be affected by respiration<br>Surrogate measures requiring validation of pathophysiological correlates<br>Inherent methodological problems: lack of input function therefore only semiquantitative |
| MMCM MRI | Blood volume<br>Permeability surface area (PS) | Blood volume<br>Vessel permeability | 'Purer' pathophysiological correlation | Limited to animal studies |
| BOLD MRI | Image intensity change<br>Change with carbogen inhalation | Vessel density<br>Blood oxygenation<br>Blood volume<br>Blood flow | No contrast agents required | Exact pathophysiological correlates unclear<br>Carbogen required |
| MRS | $^{1}H$, $^{13}C$ and $^{31}P$ spectra | Tissue bioenergetics<br>PH<br>Hypoxia<br>Oxygenation | Serial measurements possible<br>Non-invasive | Poor sensitivity |
| Doppler U/S techniques | Pulsatile index (PI)<br>Resistive index (RI)<br>Acceleration index (AI)<br>Peak systolic flow velocity ($V_{max}$)<br>Patterns of flow | Global 'flow'<br>Vascular resistance<br>Vascularity | Availability<br>Non-invasive<br>Easily repeatable<br>Low cost | Unable to image microvasculature<br>Co-registration difficulties<br>No signal at low flow |
| CT | Peak gradient in tissue<br>Peak gradient in artery | Perfusion | Availability<br>Quantitative | Single section only<br>Some methodology problems |
| Positron emission tomography;<br>PET | Perfusion<br>Blood volume<br>Specific vascular markers | Perfusion<br>Blood volume | Quantitative<br>Repeatable | Expensive<br>Limited availability |

© 2001 Cancer Research Campaign technique to measure tissue perfusion. The quantification of perfusion using dynamic computed tomography takes as its assumption that during the initial pass of the tracer through an organ there exists a time, before the tracer reaches the venous drainage, when all the tracer is within the region of interest and can be considered to be totally extracted (Hermans et al, 1997). The data acquisition time is presumed short enough that no venous outflow or extravasation of contrast has had time to occur (Blomley et al, 1993). Perfusion can thus be calculated from the equation:

$$\text{Perfusion} = \frac{\text{Peak gradient of tissue time-density curve}}{\text{Peak arterial density}}$$

(Blomley et al, 1993)

This technique has been used to calculate perfusion in the kidney, liver, pancreas and spleen (Blomley et al, 1993; Miles et al, 1998) and values obtained are similar to those obtained from other techniques such as gas washout. It has also been used to quantify perfusion in tumours (Brix et al, 1991; Hermans et al, 1997; Miles et al, 1998). It has the advantages over dynamic contrast-enhanced MRI and shares with PET the inclusion of an input function allowing accurate calculations. CT can also be used to measure indices of permeability such as contrast clearance using Patlak graphical analysis and other methods (Harvey et al, 2001).

### *Limitations*

1. Currently the technique uses high doses of radiation.
2. Currently only one section can be obtained in each scan with sufficiently high temporal resolution. Given the heterogeneity in tumours a single section may not fully represent the whole tumour. However, this is changing, with new generation scanners with detector systems allowing multiple acquisitions becoming more widely available.
3. A large vessel is necessary to determine the input function and this may not be present in the section.
4. The initial assumption that there is no venous outflow or extravasation may not hold true in tumours. Tumour vessels are characteristically more leaky than vessels in normal tissues and significant AV shunts exist.

### *Summary*

This is a promising technique but more work needs to be done in validation and application.

### CONCLUSIONS

A number of imaging modalities are available to image a range of physiological changes in tumour vasculature (see Table 1). These methods are at various stages of validation and methodology development. They all measure a variety of aspects of tumour physiology to varying degrees. The goals of the next few years will be to define exactly what pathophysiological endpoints we need to measure for what anticancer agent and to refine our ability to measure them with the imaging tools available. We need more data on the validation, reproducibility of the techniques and standardised, quantitative parameters. Experience is accumulating but more work is required.

### ACKNOWLEDGEMENTS

We would like to thank Professor John Griffiths and Professor Terry Jones for their critical review of the manuscript.

### REFERENCES

Anderson H, Yap J and Price P (2000) Measurement of tumour and normal tissue (NT) perfusion by positron emission tomography (PET) in the evaluation of antivascular therapy: Results in the Phase I study of Combretastatin A4 Phosphate (CA4P). *Proceedings of ASCO* **19**: 695(Abstract)

Araujo LI, Lammertsma AA, Rhodes CG, McFalls EO, Iida H, Rechavia E, Galassi A, de Silva R, Jones T and Maseri A (1991) Noninvasive quantification of regional myocardial blood flow in coronary artery disease with oxygen-15-labeled carbon dioxide inhalation and positron emission tomography. *Circulation* **83**: 875–885

Aronen HJ, Gazit IE, Louis DN, Buchbinder BR, Pardo FS, Weisskoff RM, Harsh GR, Cosgrove GR, Halpern EF and Hochberg FH (1994) Cerebral blood volume maps of gliomas: comparison with tumor grade and histologic findings. *Radiology* **191**: 41–51

Atkinson D, Brant-Zawadzki M, Gillan G, Purdy D and Laub G (1994) Improved MR angiography: magnetization transfer suppression with variable flip angle excitation and increased resolution. *Radiology* **190**: 890–894

Axel L (1986) Blood flow effects in magnetic resonance imaging. *Magn Reson Annu* 237–244

Blomley MJ, Coulden R, Bufkin C, Lipton MJ and Dawson P (1993) Contrast bolus dynamic computed tomography for the measurement of solid organ perfusion. *Invest Radiol* **8** (Suppl 5**)**: S72–S77

Boetes C, Barentsz JO, Mus RD, van der Sluis RF, van Erning LJ, Hendriks JH, Holland R and Ruys SH (1994) MR characterization of suspicious breast lesions with a gadolinium-enhanced TurboFLASH subtraction technique. *Radiology* **193**: 777–781

Boxerman JL, Hamberg LM, Rosen BR and Weisskoff RM (1995) MR contrast due to intravascular magnetic susceptibility perturbations. *Magn Reson Med* **34**: 555–566

Brasch R, Pham C, Shames D, Roberts T, van Dijke K, van Bruggen N, Mann J, Ostrowitzki S and Melnyk O (1997) Assessing tumor angiogenesis using macromolecular MR imaging contrast media. *J Magn Reson Imaging* **7**: 68–74

Brix G, Semmler W, Port R, Schad LR, Layer G and Lorenz WJ (1991) Pharmacokinetic parameters in CNS Gd-DTPA enhanced MR imaging. *J Comput Assist Tomogr* **15**: 621–628

Burns PN, Halliwell M, Wells PN and Webb AJ (1982) Ultrasonic Doppler studies of the breast. *Ultrasound Med Biol* **8**: 127–143

Crew JP, O'Brien T, Bicknell R, Fuggle S, Cranston D and Harris AL (1999) Urinary vascular endothelial growth factor and its correlation with bladder cancer recurrence rates [see comments]. *J Urol* **161**: 799–804

Davies MM, Jonas SK, Kaur S and Allen-Mersh TG (2000) Plasma vascular endothelial but not fibroblast growth factor levels correlate with colorectal liver mestastasis vascularity and volume. *British Journal of Cancer* **82**: 1004–1008

Delorme S and Knopp MV (1998) Non-invasive vascular imaging: assessing tumour vascularity. *Eur Radiol* **8**: 517–527

Dumoulin CL and Hart HRJ (1986) Magnetic resonance angiography. *Radiology* **161**: 717–720

Emoto M, Iwasaki H, Mimura K, Kawarabayashi T and Kikuchi M (1997) Differences in the angiogenesis of benign and malignant ovarian tumors, demonstrated by analyses of color Doppler ultrasound, immunohistochemistry, and microvessel density. *Cancer* **80**: 899–907

Ferrara N (1996) Vascular endothelial growth factor. *European Journal of Cancer* 32A: 2413–2422

Fisel CR, Ackerman JL, Buxton RB, Garrido L, Belliveau JW, Rosen BR and Brady TJ (1991) MR contrast due to microscopically heterogeneous magnetic susceptibility: numerical simulations and applications to cerebral physiology. *Magn Reson Med* **17**: 336–347

Fobben ES, Rubin CZ, Kalisher L, Dembner AG, Seltzer MH and Santoro EJ (1995) Breast MR imaging with commercially available techniques: radiologic-pathologic correlation. *Radiology* **196**: 143–152

Folkman J (1971) Tumor angiogenesis: therapeutic implications. *N Engl J Med* **285**: 1182–1186

Gasparini G (1996) Clinical significance of the determination of angiogenesis in human breast cancer: update of the biological background and overview of the Vicenza studies. *Eur J Cancer* **32A**: 2485–2493

Harvey CJ, Blomley MJ, Eckersley RJ, Heckemann RA, Butler-Barnes J and Cosgrove DO (2000) Pulse-inversion mode imaging of liver specific

*© 2001 Cancer Research Campaign*

microbubbles: improved detection of subcentimetre metastases [letter]. *Lancet* **355**: 807–808
Harvey CJ, Blomley MJ, Dawson P et al. Functional CT images of the acute hyperemic response to radiation of the prostate gland: early experience. *J Comput Assist Tomogr* **25**: 43–49
Hawighorst H, Knapstein PG, Knopp MV, Weikel W, Brix G, Zuna I, Schonberg SO, Essig M, Vaupel P and van Kaick G (1998) Uterine cervical carcinoma: comparison of standard and pharmacokinetic analysis of time-intensity curves for assessment of tumor angiogenesis and patient survival. *Cancer Res* **58**: 3598–3602
Hermans R, Lambin P, Van den Bogaert W, Haustermans K, Van der Goten A and Baert AL (1997) Non-invasive tumour perfusion measurement by dynamic CT: preliminary results. *Radiother Oncol* **44**: 159–162
Heywang SH, Wolf A, Pruss E, Hilbertz T, Eiermann W and Permanetter W (1989) MR imaging of the breast with Gd-DTPA: use and limitations. *Radiology* **171**: 95–103
Hoffmann U, Brix G, Knopp MV, Hess T and Lorenz WJ (1995) Pharmacokinetic mapping of the breast: a new method for dynamic MR mammography. *Magn Reson Med* **33**: 506–514
Horsman MR, Ehrnrooth E, Ladekarl M and Overgaard J (1998) The effect of combretastatin A-4 disodium phosphate in a C3H mouse mammary carcinoma and a variety of murine spontaneous tumors. *Int J Radiat Oncol Biol Phys* **42**: 895–898
Haubner R et al (2001) Non invasive imaging of $\alpha_v\beta_3$ integrin expression using1 $^{18}$F-labeled RGD-containing glycopeptide and positron emission tomography. *Cancer Res* **61**: 1781–1785
Hulka CA, Edmister WB, Smith BL, Tan L, Sgroi DC, Campbell T, Kopans DB and Weisskoff RM (1997) Dynamic echo-planar imaging of the breast: experience in diagnosing breast carcinoma and correlation with tumor angiogenesis. *Radiology* **205**: 837–842
Jain RK (1988) Determinants of tumor blood flow: a review. *Cancer Res* **48**: 2641–2658
Kaiser WA (1993) [MR mammography] MR-Mammographie. *Radiologe* **33**: 292–299
Kaiser WA (1994) False-positive results in dynamic MR mammography. Causes, frequency, and methods to avoid. *Magn Reson Imaging Clin N Am* **2**: 539–555
Karczmar GS, River JN, Li J, Vijayakumar S, Goldman Z and Lewis MZ (1994) Effects of hyperoxia on T2* and resonance frequency weighted magnetic resonance images of rodent tumours. *NMR Biomed* **7**: 3–11
Kerslake RW, Carleton PJ, Fox JN, Imrie MJ, Cook AM, Read JR, Bowsley SJ, Buckley DL and Horsman A (1995) Dynamic gradient-echo and fat-suppressed spin-echo contrast-enhanced MRI of the breast [see comments]. *Clin Radiol* **50**: 440–454
Koh WJ, Rasey JS, Evans ML, Grierson JR, Lewellen TK, Graham MM, Krohn KA and Griffin TW (1992) Imaging of hypoxia in human tumors with [F-18]fluoromisonidazole. *Int J Radiat Oncol Biol Phys* **22**: 199–212
Koh WJ, Bergman KS, Rasey JS, Peterson LM, Evans ML, Graham MM, Grierson JR, Lindsley KL, Lewellen TK and Krohn KA (1995) Evaluation of oxygenation status during fractionated radiotherapy in human nonsmall cell lung cancers using [F-18]fluoromisonidazole positron emission tomography. *Int J Radiat Oncol Biol Phys* **33**: 391–398
Konig H, Sieper J and Wolf KJ (1990) Rheumatoid arthritis: evaluation of hypervascular and fibrous pannus with dynamic MR imaging enhanced with Gd-DTPA. *Radiology* **176**: 473–477
Kouwenhoven M (1997) Contrast-enhanced MR angiography. Methods, limitations and possibilities. *Acta Radiol Suppl* **412**: 57–67
Kuwatsuru R, Shames DM, Muhler A, Mintorovitch J, Vexler V, Mann JS, Cohn F, Price D, Huberty J and Brasch RC (1993) Quantification of tissue plasma volume in the rat by contrast-enhanced magnetic resonance imaging. *Magn Reson Med* **30**: 76–81
Lammertsma AA, Wise RJ and Jones T (1983) In vivo measurements of regional cerebral blood flow and blood volume in patients with brain tumours using positron emission tomography. *Acta Neurochir (Wien)* **69**: 5–13
Lammertsma AA, Cunningham VJ, Deiber MP, Heather JD, Bloomfield PM, Nutt J, Frackowiak RS and Jones T (1990) Combination of dynamic and integral methods for generating reproducible functional CBF images. *J Cereb Blood Flow Metab* **10**: 675–686
Leenders KL (1994) PET: blood flow and oxygen consumption in brain tumors. *J Neurooncol* **22**: 269–273
Lindsay CK, Gomez DE and Thorgeirsson UP (1996) Effect of flavone acetic acid on endothelial cell proliferation: evidence for antiangiogenic properties. *Anticancer Res* **16**: 425–431
Martinoli C, Derchi LE, Rizzato G and Solbiati L (1998) Power Doppler sonography: general principles, clinical applications, and future prospects. *Eur Radiol* **8**: 1224–1235
Mayr NA, Yuh WT, Magnotta VA, Ehrhardt JC, Wheeler JA, Sorosky JI, Davis CS, Wen BC, Martin DD, Pelsang RE, Buller RE, Oberley LW, Mellenberg DE and Hussey DH (1996) Tumor perfusion studies using fast magnetic resonance imaging technique in advanced cervical cancer: a new noninvasive predictive assay. *Int J Radiat Oncol Biol Phys* **36**: 623–633
McCoy CL, Parkins CS, Chaplin DJ, Griffiths JR, Rodrigues LM and Stubbs M (1995) The effect of blood flow modification on intra-and extracellular pH measured by 31P magnetic resonance spectroscopy in murine tumours. *Br J Cancer* **72**: 905–911
Miles KA, Leggett DA, Kelley BB, Hayball MP, Sinnatamby R and Bunce I (1998) In vivo assessment of neovascularization of liver metastases using perfusion CT [see comments]. *Br J Radiol* **71**: 276–281
Mountford RA and Atkinson P (1979) Doppler ultrasound examination of pathologically enlarged lymph nodes. *Br J Radiol* **52**: 464–467
Nielsen FU, Topp S, Horsman MR, Overgaard J, Stodkilde-Jorgensen H and Maxwell RJ (1999) Localized in vivo 1H NMR spectroscopy of murine tumours: effect of blood flow reduction. *NMR Biomed* **12**: 175–183
O'Reilly MS (1997) Angiostatin: an endogenous inhibitor of angiogenesis and of tumor growth. *EXS* **79**: 273–294
Ogawa S, Lee TM, Kay AR and Tank DW (1990) Brain magnetic resonance imaging with contrast dependent on blood oxygenation. *Proc Natl Acad Sci USA* **87**: 9868–9872
Orel SG, Schnall MD, LiVolsi VA and Troupin RH (1994) Suspicious breast lesions: MR imaging with radiologic-pathologic correlation. *Radiology* **190**: 485–493
Parker GJ, Suckling J, Tanner SF, Padhani AR, Revell PB, Husband JE and Leach MO (1997) Probing tumor microvascularity by measurement, analysis and display of contrast agent uptake kinetics. *J Magn Reson Imaging* **7**: 564–574
Perman WH, Heiberg EV and Herrmann VM (1996) Half-Fourier, three-dimensional technique for dynamic contrast-enhanced MR imaging of both breasts and axillae: initial characterization of breast lesions. *Radiology* **200**: 263–269
Peters-Engl C, Medl M, Mirau M, Wanner C, Bilgi S, Sevelda P and Obermair A (1998) Color-coded and spectral Doppler flow in breast carcinomas – relationship with the tumor microvasculature. *Breast Cancer Res Treat* **47**: 83–89
Postema S, Pattynama PM, Broker S, van der Geest RJ, van Rijswijk CS and Baptist TJ (1998) Fast dynamic contrast-enhanced colour-coded MRI in uterine cervix carcinoma: useful for tumour staging? *Clin Radiol* **53**: 729–734
Rasey JS, Koh WJ, Evans ML, Peterson LM, Lewellen TK, Graham MM and Krohn KA (1996) Quantifying regional hypoxia in human tumors with positron emission tomography of [18F] fluoromisonidazole: a pretherapy study of 37 patients. *Int J Radiat Oncol Biol Phys* **36**: 417–428
Reeder SB, Atalay MK, McVeigh ER, Zerhouni EA and Forder JR (1996) Quantitative cardiac perfusion: a noninvasive spin-labeling method that exploits coronary vessel geometry. *Radiology* **200**: 177–184
Roberts TP (1997) Physiologic measurements by contrast-enhanced MR imaging: expectations and limitations. *J Magn Reson Imaging* **7**: 82–90
Robinson SP, Howe FA and Griffiths JR (1995) Noninvasive monitoring of carbogen-induced changes in tumor blood flow and oxygenation by functional magnetic resonance imaging [see comments]. *Int J Radiat Oncol Biol Phys* **33**: 855–859
Robinson SP, Howe FA, Stubbs M and Griffiths JR (2000) Effects of nicotinamide and carbogen on tumour oxygenation, blood flow, energetics and blood glucose levels. *Br J Cancer* **82**: 2007–2014
Rosen BR, Belliveau JW, Vevea JM and Brady TJ (1990) Perfusion imaging with NMR contrast agents. *Magn Reson Med* **14**: 249–265
Shames DM, Kuwatsuru R, Vexler V, Muhler A and Brasch RC (1993) Measurement of capillary permeability to macromolecules by dynamic magnetic resonance imaging: a quantitative noninvasive technique. *Magn Reson Med* **29**: 616–622
Sterns EE, SenGupta S, Saunders F and Zee B (1996) Vascularity demonstrated by Doppler ultrasound and immunohistochemistry in invasive ductal carcinoma of the breast. *Breast Cancer Res Treat* **40**: 197–203
Stubbs M (1999) Application of magnetic resonance techniques for imaging tumour physiology. *Acta Oncol* **38**: 845–853
Su MY, Jao JC and Nalcioglu O (1994) Measurement of vascular volume fraction and blood-tissue permeability constants with a pharmacokinetic model: studies in rat muscle tumors with dynamic Gd-DTPA enhanced MRI. *Magn Reson Med* **32**: 714–724
Tofts PS and Kermode AG (1991) Measurement of the blood-brain barrier permeability and leakage space using dynamic MR imaging. 1. Fundamental concepts. *Magn Reson Med* **17**: 357–367
Toi M, Hoshina S, Takayanagi T and Tominaga T (1994) Association of vascular endothelial growth factor expression with tumor angiogenesis and with early

 *© 2001 Cancer Research Campaign*

relapse in primary breast cancer. *Japanese Journal of Cancer Research* **85**: 1045–1049

van Zijl PC, Eleff SM, Ulatowski JA, Oja JM, Ulug AM, Traystman RJ and Kauppinen RA (1998) Quantitative assessment of blood flow, blood volume and blood oxygenation effects in functional magnetic resonance imaging [see comments]. *Nat Med* **4**: 159–167

Verstraete KL, De Deene Y, Roels H, Dierick A, Uyttendaele D and Kunnen M (1994) Benign and malignant musculoskeletal lesions: dynamic contrast-enhanced MR imaging–parametric "first-pass" images depict tissue vascularization and perfusion. *Radiology* **192**: 835–843

Villringer A, Rosen BR, Belliveau JW, Ackerman JL, Lauffer RB, Buxton RB, Chao YS, Wedeen VJ and Brady TJ (1988) Dynamic imaging with lanthanide chelates in normal brain: contrast due to magnetic susceptibility effects. *Magn Reson Med* **6**: 164–174

Wamil BD, Thurman GB, Sundell HW, DeVore RF, Wakefield G, Johnson DH, Wang YF and Hellerqvist CG (1997) Soluble E-selectin in cancer patients as a marker of the therapeutic efficacy of CM101, a tumor-inhibiting anti-neovascularization agent, evaluated in phase I clinical trial. *J Cancer Res Clin Oncol* **123**: 173–179

Watson SA, Morris TM, Parsons SL, Steele RJ and Brown PD (1996) Therapeutic effect of the matrix metalloproteinase inhibitor, batimastat, in a human colorectal cancer ascites model. *Br J Cancer* **74**: 1354–1358

Wedeen VJ, Meuli RA, Edelman RR, Geller SC, Frank LR, Brady TJ and Rosen BR (1985) Projective imaging of pulsatile flow with magnetic resonance. *Science* **230**: 946–948

Wei K, Jayaweera AR, Firoozan S, Linka A, Skyba DM and Kaul S (1998) Quantification of myocardial blood flow with ultrasound-induced destruction of microbubbles administered as a constant venous infusion. *Circulation* **97**: 473–483

Weissleder R, Cheng HC, Marecos E, Kwong K and Bogdanov AJ (1998) Non-invasive in vivo mapping of tumour vascular and interstitial volume fractions. *Eur J Cancer* **34**: 1448–1454

Wells PT, Halliwell M, Skidmore R, Webb AJ and Woodcock JP (1977) Tumour detection by ultrasonic Doppler blood-flow signals. *Ultrasonics* **15**: 231–232

White DN and Cledgett PR (1978) Breast carcinoma detection by ultrasonic Doppler signals. *Ultrasound Med Biol* **4**: 329–335

Wilson CB, Lammertsma AA, McKenzie CG, Sikora K and Jones T (1992) Measurements of blood flow and exchanging water space in breast tumors using positron emission tomography: A rapid and noninvasive dynamic Method. *Cancer Res* **52**: 1592–1597

© 2001 Cancer Research Campaign

